# The case for inhibiting p38 mitogen-activated protein kinase in heart failure

**DOI:** 10.3389/fphar.2015.00102

**Published:** 2015-05-12

**Authors:** Pelin Arabacilar, Michael Marber

**Affiliations:** Cardiovascular Division, Department of Cardiology, King's College London British Heart Foundation Centre, The Rayne Institute, St Thomas' HospitalLondon, UK

**Keywords:** p38, MAPK, heart failure, hypertrophy, fibrosis, inflammation

## Abstract

This minireview discusses the evidence that the inhibition of p38 mitogen-activated protein kinases (p38 MAPKs) maybe of therapeutic value in heart failure. Most previous experimental studies, as well as past and ongoing clinical trials, have focussed on the role of p38 MAPKs in myocardial infarction and acute coronary syndromes. There is now growing evidence that these kinases are activated within the myocardium of the failing human heart and in the heart and blood vessels of animal models of heart failure. Furthermore, from a philosophical viewpoint the chronic activation of the adaptive stress pathways that lead to the activation of p38 MAPKs in heart failure is analogous to the chronic activation of the sympathetic, renin-aldosterone-angiotensin and neprilysin systems. These have provided some of the most effective therapies for heart failure. This minireview questions whether similar and synergistic advantages would follow the inhibition of p38 MAPKs.

## Introduction to heart failure

Heart failure, also referred to as chronic or congestive heart failure, is a progressive condition which occurs when the heart at a normal filling pressure is unable to pump sufficient blood to meet the body's requirements. The syndrome is multifaceted including abnormities in heart muscle, valves and/or pericardium as well as systemic disturbances in neuro-humoral, cytokine and/or vascular function (Marks, 2013). The changes can include but are not limited to deterioration in the force of contraction and vascular tone and alterations in hypertrophy, apoptosis, fibrosis, autophagy and inflammatory cytokines as will be discussed further in this review.

## Evidence for p38 activation in heart failure

**Figure 1:**
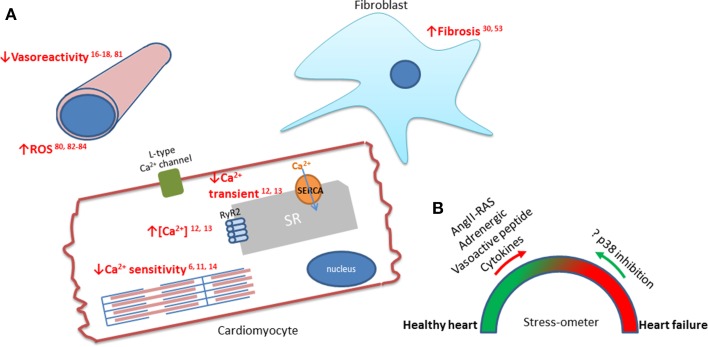
**The downstream effects of p38 in cardiomyocytes, fibroblasts, and vasculature during heart failure. (A)** Emphasized in red are the effects of p38 which are associated with the progression of heart failure. p38 activity has been linked to increased interstitial fibrosis (Wang et al., 1998; Ma et al., 1999), reduced vasoreactivity (Behr et al., 2001; Vijayan et al., 2004; Kumar et al., 2008; Hoefer et al., 2010) and increased ROS production (Li et al., 2005; Aukrust et al., 2011; Denise Martin et al., 2012; Elkhawad et al., 2012). The effects on the force of cardiomyocyte contraction are mediated by the effect of p38 on the Ca^2+^ transient (Andrews et al., 2003; Kaikkonen et al., 2014) and the sensitivity of the sarcomeres (Liao et al., 2002; Kan et al., 2004; Vahebi et al., 2007). **(B)** Schematic showing balance between stresses on the heart that lead to healthy adaptation and the pathological increases in cytokines and neurohormones that lead to, or aggravate, heart failure. The question is whether these pathological signals can be reversed by inhibiting p38?

The mitogen-activated protein kinase p38 is a key Ser/Thr kinase that responds to a variety of the multifaceted abnormalities contributing to heart failure (see Figure 1). There have been extensive studies on the role of p38 in different disease states, primarily ischaemic heart disease in the cardiac setting. Though not as thoroughly explored as ischaemia, the role of p38 has been investigated in heart failure. Activation of p38 has been observed in animal models of heart failure and studies on myocardial biopsies from heart failure patients show increased p38 activity in comparison to “healthy” hearts (Takeishi et al., 2002; Ng et al., 2003; Bellahcene et al., 2006). In cultured cardiomyocytes, p38 activation augments hypertrophy and pharmacologic inhibition attenuates hypertrophy occurring in response to stimuli such as endothelin-1 and phenylephrine (Nemoto et al., 1998). Whilst inhibiting p38 activity using SB203580 in adult rat cardiomyocytes, increases contractility (Liao et al., 2002). Variations in patient populations and genetic differences between animal models make it challenging to determine the precise role of p38 in heart failure. Collectively, it appears that p38 plays an important role in the progression of heart failure. The structure and function of p38 has been recently reviewed in the cardioprotective context (Martin et al., 2015). In this review, we will elucidate the mechanisms and consequences of p38 activity in heart failure with the aim of highlighting areas for further research required to clarify future potential therapeutic benefit.

## Pathological features of heart failure that may lie downstream of p38

### The force of contraction

An intricate protein signaling cascade exists to control the contraction of cardiomyocytes. The increase in cytosolic Ca^2+^ ions in the cell leads to actin interacting with myosin and the power stroke that shortens the sarcomere. Most heart failure is characterized by decreased contractility and reduced ejection fraction and the most common underlying process is pressure overload due to hypertension or cavity dilatation after myocardial infarction acting through the Law of LaPlace. Pressure overload in turn leads to hypertrophy and loss of contractile function (Peterson, 2002; Lips et al., 2003). It appears that activation of the p38 pathway depresses contractility and enhances matrix remodeling (Kerkela and Force, 2006). In studies involving the activation of this pathway through gene transfer of the activated upstream kinases of p38 [mitogen-activated protein kinase kinase 3/6 (MKK3/6)] or blocking the pathway through dominant negative p38 mutants and pharmacologic inhibition, it is evident that p38 activity leads to negative inotropic effects (Liao et al., 2002; Vahebi et al., 2007). More than one mechanism has been proposed through which p38 decreases contractility. Such mechanisms include prolongation of the decay phase of the cardiac calcium transient increasing diastolic Ca^2+^ concentration and relaxation. This is thought to be mediated through downregulation of sarcoplasmic/endoplasmic reticulum calcium ATPase (SERCA2), responsible for the translocation of Ca^2+^ from the cytosol to the sarcoplasmic reticulum (Andrews et al., 2003; Kaikkonen et al., 2014). In support of this, tumor necrosis factor α (TNFα)-induced contractile dysfunction in isolated hearts is attenuated in MKK3 knockout mice and also by pharmacologic inhibition (Bellahcene et al., 2006). Furthermore, other studies suggest that there may be an additional contribution through p38 activity diminishing the Ca^2+^ sensitivity of the sarcomere (Liao et al., 2002; Kan et al., 2004; Vahebi et al., 2007).

### Vascular tone

p38 is activated in the vessel wall in response to pressure overload, hypoxia and heart failure (Kyriakis and Avruch, 2001; Hoefer et al., 2010) and also by neurohormonal stimuli such as angiotensin II or endothelin-1; both associated with vasoconstriction and ventricular remodeling (Vijayan et al., 2004; Kumar et al., 2008). Vasoreactivity is improved, and survival is increased, by pharmacologic inhibition of p38 in several different models involving these stressors (See et al., 2004; Bao et al., 2007; Hoefer et al., 2010). In a rat model of heart failure, inhibition of p38 with SB239063 normalizes vascular p38 activity and endothelial dysfunction is prevented (Widder et al., 2004). Current literature mainly focuses on the activation mechanisms of p38 during heart failure and less on the downstream mechanisms which may lead to the pathological features of the syndrome. Though further investigation is required, it appears that SB239063 leads to a decrease in vascular superoxide anion formation, suggesting that p38 plays a role in generation of reactive oxygen species (ROS) during heart failure (Widder et al., 2004). Furthermore, one of the main subsets of p38 substrates are transcription factors, such as myocyte enhancer factor 2A (MEF2A) and myocyte enhancer factor 2C (MEF2C), that are implicated in the regulation of vascular tone (Wang et al., 2003; Hayashi et al., 2004; Olson, 2004), as well as ROS generation. In summary, the dysfunction mediated in part by p38 appears to be via ROS generation and possibly an effect on downstream transcription factors (Ushio-Fukai et al., 1998; Li et al., 2004; Bao et al., 2007).

### Hypertrophy

The data concerning the role of p38 in hypertrophy are difficult to reconcile; there is evidence both for and against its involvement (Haq et al., 1998; Nishida et al., 2004; Chahine et al., 2015). Perhaps due to differences in models and experimental detail, substantial variability of p38 activity in heart failure and/or hypertrophy has been observed.

In an adenoviral-mediated overexpression system in cardiomyocytes expressing upstream activators for p38; MKK3 and MKK6, leads to a pro-hypertrophic response including increase in cell size and atrial natriuretic factor expression, suggesting a causative role (Wang et al., 1998). However, in an *in vivo* model with transgenic mice expressing the dominant-negative mutants of MKK3, MKK6, and p38α, cardiac hypertrophy following aortic banding is enhanced, potentially through the regulation of nuclear factor of activated T cells (NFAT) (Braz et al., 2003). *In vivo* studies, and data acquired from heart failure patients, suggest that p38 contributes to the progression of heart failure but that this is not through the aggravation of hypertrophy (Ng et al., 2003; Nishida et al., 2004; See et al., 2004; Klein et al., 2005). For example, p38 does not appear to be activated in hypertrophied hearts, but in failing hearts a two-fold increase in p38 phosphorylation is observed (Haq et al., 1998).

Overall, in isolated cardiomyocytes p38 activation appears to increase hypertrophy and its inhibition, using pharmacological compounds or genetic methods, attenuates the development of hypertrophy in response to hypertrophic stimuli (Nemoto et al., 1998; Wang et al., 1998; Liang and Molkentin, 2003). However, the picture is more complex in *in vivo* models and it is not clear that hypertrophy in the absence of heart failure causes p38 activation in patients.

### Apoptosis

Cardiomyocyte death is an integral component of decompensated cardiac hypertrophy and the dysfunction leading to heart failure (Diwan et al., 2008). Three systems of cell death exist, namely; necrosis, apoptosis and autophagy. Cardiac apoptosis is regulated by an elaborate array of stress-activated signaling pathways. p38 has been associated with both anti- and pro-apoptotic downstream effects depending on the upstream stimulus and cell-type (Chuang et al., 2000; Okamoto et al., 2000; Kaiser et al., 2004; Kilpatrick et al., 2006). However, in the cardiac setting, the role of p38 in regulating apoptosis is still under investigation. The apoptotic effects of anisomycin and overexpressing activated mitogen-activated protein kinase kinase 1(MEKK1) are reversed by overexpressing constitutively active MKK6 (Zechner et al., 1998) and a similar result is observed with the augmentation of norepinephrine-induced apoptosis by a p38 inhibitor in cardiac myocytes (Communal et al., 2000). It appears that the protective role of MKK6 overexpression is, in part, through nuclear factor κB (NFκB) activation, interleukin 6 (IL-6) induction and αB-crystallin phosphorylation (Zechner et al., 1998; Craig et al., 2000; Hoover et al., 2000; Zhao et al., 2013).

Nonetheless, there are a few reports contradicting these findings, suggesting that p38 activation is, in fact, pro-apoptotic in cardiomyocytes. In transgenic mice with cardiac-specific expression of a dominant-negative mutant form of p38α after experimental diabetes; myocardial apoptosis, the number of caspase-3-positive cells, and the downregulation of antiapoptotic protein B-cell lymphoma-extra large (Bcl-X_L_) are all attenuated, suggesting a pro-apoptotic role for p38 (Thandavarayan et al., 2009). In addition, it has been previously reported that apoptosis is reduced by p38 inhibitors; SB203580, SB239063, or FR167653 in cardiac cells in response to several stimuli (Mackay and Mochly-Rosen, 1999, 2000; Zhu et al., 1999; Kang et al., 2000; Sharov et al., 2003; Kyoi et al., 2006). In isolated perfused hearts, p38 inhibitors are also cardioprotective (Meldrum et al., 1998; Ma et al., 1999; Barancik et al., 2000). In bovine aorta endothelial cells, p38 involvement on β_2_AR-mediated caspase-3 cleavage is suggested via negative regulation by the p38 inhibitor SB203580 (Iaccarino et al., 2005). In Raf-1-knockout mice which demonstrated left ventricular systolic dysfunction, heart dilatation and an increase in apoptosis was associated with an increase in p38 kinase activity (Yamaguchi et al., 2004). Furthermore, overexpression of p38α or activated MKK3b in cultured neonatal cardiomyocytes (Wang et al., 1998) and expression of transforming growth factor-β-activated kinase-1 (TAK1) in the mouse heart by transgenesis, are associated with increased cardiac apoptosis (Zhang et al., 2000).

The opposing findings on the role of p38 in apoptosis could be attributed to variation among genetic models and non-specific effects of pharmacologic compounds. Nonetheless, the literature in models utilizing more specific methods which are less prone to off-target effects, such as overexpression of wild-type or dominant-negative mutants, indicates that its activation plays a pro-apoptotic role in the cardiac setting.

### Fibrosis

As already discussed, in cultured cardiomyocytes p38 activity is associated with myocyte hypertrophy and apoptosis. It also appears that p38 activity in cardiomyocytes contributes to remodeling in the adult heart. In intact mouse hearts although p38 overexpression/activation does not lead to hypertrophy, it increases remodeling of the extracellular matrix and diminishes contractile function (Liao et al., 2001, 2002; Biesemann et al., 2015). In a p38α knock out mouse model exposed to pressure overload, increased interstitial fibrosis is observed (Nishida et al., 2004) whilst a mouse model expressing dominant-negative p38α displays resistance to fibrosis in response to pressure overload (Zhang et al., 2003). Furthermore, p38 activation using cre/loxP-based gene switch to create transgenic animals expressing the activated upstream kinases of p38, MKK3b, and MKK6, leads to the induction of interstitial fibrosis, depresses contractility and compromises diastolic function (Liao et al., 2001).

In a recent study, overexpression of myostatin, a member of the TGF-β superfamily that is up-regulated under disease conditions, is shown to cause interstitial fibrosis via activation of the TAK1-MKK3/6-p38 pathway suggesting that upstream effectors also play a role in p38 activation leading to fibrosis. In addition, mitogen activated protein kinase-activated protein kinase 2 (MK2) is an important substrate of p38 that is associated with heart failure; since cardiac fibrosis and dysfunction are diminished in MK2 knockout mice (Streicher et al., 2010; Scharf et al., 2013) potentially through an involvement of SERCA2 regulation. Treatment of hamster hearts with SB203580 reduces the area of fibrosis and heart /body weight ratio, increases LV ejection fraction and contractility (Kyoi et al., 2006). These findings provide evidence that p38 activation can contribute to fibrosis in the failing heart.

### Autophagy

Autophagy serves as a double-edged sword with both anti and pro-apoptotic functions. In patients with heart failure, an increase in autophagy is observed and is associated with left ventricular systolic dysfunction (Hein et al., 2003; Vigliano et al., 2011). There are studies suggesting that autophagy is a maladaptive process during the progression of heart failure and others which propose a protective role. *In vivo* studies blunting autophagy using 3-metyladenine, an inhibitor of class III phosphoinositide-3-kinase (PI3K), show heart failure progression is accelerated with an increase in interstitial fibrosis, worsening ventricular function and early mortality (Tannous et al., 2008). In addition, a decrease in autophagy has been implicated in cardiac hypertrophy whilst an increase in autophagy in transgenic mouse models has been linked to cardio-protection (Ceylan-Isik et al., 2013). However, excessive autophagy has been associated with the progression of cardiac remodeling and heart failure in response to pressure overload (Nakai et al., 2007). Molecular studies of biopsy samples of left ventricular myocardium from patients with idiopathic dilated cardiomyopathy before the implantation, and after the removal, of a left ventricular assist device suggest that mechanical unloading of the heart leads to a decrease in markers of autophagy (Kassiotis et al., 2009). In this study, it is suggested that autophagy may serve an adaptive purpose during the progression of heart failure.

Autophagy-related genes are upregulated in response to H_2_O_2_ treatment in myotubes, with a positive correlation with p38 activation. Inhibition of p38 using SB202190 decreases H_2_O_2_-induced expression of Atg7 (McClung et al., 2010). However, in senescent human CD8^+^ T cells, p38 inhibition using BIRB796 inhibits autophagy (Henson et al., 2014). In addition, in cultured neonatal rat cardiomyocytes exposed to 48 h of mechanical stretch and in mice following transverse aortic constriction, p38 inhibition causes a decrease in the autophagy marker microtubule associated protein 1 light chain 3 β II (LC3b-II) (Lin et al., 2014). As the impact of autophagy in heart failure itself is controversial, it is difficult to assess whether the effect of p38 activation is protective or detrimental in heart failure. Nonetheless, it is apparent that p38 plays a role in the mechanism of autophagy.

### Inflammation and cytokine signaling

Increasing evidence indicates that inflammatory cytokines, including TNF-α, interleukin 1β (IL-1β), and IL-6, are elevated in, and may contribute to, heart failure. TNF-α levels increase in patients with advanced heart failure and correlate with prognosis (Levine et al., 1990; Feldman et al., 2000; Behnam et al., 2005; Gong et al., 2007). TNF-α is not expressed in the non-failing heart, but is significantly increased in the end stage failing human hearts (Torre-Amione et al., 1996). This has been associated with negative inotropic effects, with the IL-1β-mediated expression of SERCA and phospholamban prolonging the Ca^2+^ transient (McTiernan et al., 1997; Feldman et al., 2000). In addition, TNF-α induction in failing hearts leads to a further loss of contractility and worsening of extracellular matrix remodeling (Yokoyama et al., 1997; Sivasubramanian et al., 2001). Interestingly, a similar array of pathological alterations is observed in response to p38 activation (Liao et al., 2001). Due to more than one inflammatory cytokine being associated with the progression of heart failure, studies involving manipulation of only one factor might not be the most effective way to investigate their summative effect on heart failure. Thus, p38, by regulating varied inflammatory cytokines, becomes a more attractive therapeutic target and consequently has been explored in a number of studies (Marber et al., 2011; Denise Martin et al., 2012). In SB239063-treated spontaneously hypertensive stroke-prone rats, pro-inflammatory gene expression is attenuated and survival is increased (Behr et al., 2001). In transgenic mice expressing active MKK6, TNF-α and IL-6 induction and extracellular remodeling is increased (Li et al., 2005). Administration of SB239068 in the same transgenic model reduces plasma levels of these cytokines, interstitial fibrosis and cardiac remodeling. In a MKK3 knock out model, similar results to pharmacologic inhibition of p38 are observed with a reduction in TNF-α-induced contractile dysfunction (Bellahcene et al., 2006). Furthermore, knocking out MK2 in mice prevents the TNF-α-induced negative inotropic response (Bellahcene et al., 2006). Other than the direct induction of inflammatory cytokine production, p38 is implicated in the amplification of ROS generation; a principal feature of vascular inflammation (Hoefen and Berk, 2002; Goettsch et al., 2009; Aukrust et al., 2011; Elkhawad et al., 2012). These studies suggest that p38 inhibition in the stressed heart will be beneficial, at least in part through the suppression of inflammatory cytokines and consequent improvement in myocardial remodeling.

## Clinical trials

With the detrimental effects observed in *in vivo* and *in vitro* studies, clinical trials using the agents; etanercept, a decoy approach to block TNF-α interaction with its receptor and infliximab, monoclonal antibody to neutralize TNF-α were performed. Unfortunately, results of either no benefit or harm are observed (Mann, 2005). A clinical trial for semapimod, an anti-inflammatory agent which inhibits p38 activity, was started for heart failure patients, but was apparently terminated upon the disclosure of the discouraging results of the TNF-α-targeted clinical trials (Kerkela and Force, 2006).

Currently, there is an ongoing phase 3 clinical trial running with losmapimod (LATITUDE-TIMI 60, NCT02145468), which could potentially benefit acute coronary syndrome (ACS) patients. Losmapimod is an anti-inflammatory medication which inhibits p38 and may improve vascular function and reduce subsequent cardiac events following ACS.

Though further research is required on the mechanisms and consequences of p38 activation in heart failure, this review has discussed the substantial evidence for an important role of p38 in the development of heart failure and its potential as a therapeutic target.

### Conflict of interest statement

The authors declare that the research was conducted in the absence of any commercial or financial relationships that could be construed as a potential conflict of interest.
